# The success of opening single chronic total occlusion lesions to improve myocardialviabilitytrial (SOS-COMEDY): Study protocol of a prospective multicenter study

**DOI:** 10.1097/MD.0000000000010443

**Published:** 2018-04-20

**Authors:** Rongchong Huang, Xiantao Song, Haishan Zhang, Wen Tian, Zheng Huang, Xingwei Zhang, Junqing Yang, Dongfeng Zhang, Jian Wu, Lei Zhong, Henry H. Ting

**Affiliations:** a The Department of Cardiology, The First Affiliated Hospital of Dalian Medical University, Dalian City; b The Department of Cardiology, Beijing An Zhen Hospital, Capital Medical University, Beijing City; c The Department of Cardiology, First Affiliated Hospital of China Medical University, Shenyang City; d The Department of Cardiology, Southern Hospital, Southern Medical University, Guangzhou City; e The Department of Cardiology, The Affiliated Hospital of Hangzhou Normal University, Hangzhou City; f The Department of Cardiology, Guangdong General Hospital, Guangzhou City, China; g Division of Cardiology, Mayo Clinic, Jacksonville, FL.

**Keywords:** chronic total occlusion, coronary intervention, decision aid, noninvasive imaging

## Abstract

**Aims:**

Success of opening single (SOS)-comedy is a prospective multicenter study to compare the improvement in the decrease of myocardial viability by percutaneous coronary intervention (PCI) with that by optimal medical therapy (OMT) alone in patients with chronic total occlusion (CTO) of a single coronary artery.

**Methods and results:**

The risks and the benefits of both options (PCI and OMT) were listed in a CTO decision aid (DA). Eligible participants detected by invasive coronary angiography (ICA) or coronary computed tomography angiography (CCTA) were divided into PCI or OMT groups according to patients’ choice after shared-decision making process with DA. Participants will undergo positron emission tomography/computed tomography (PET/CT), cardiac magnetic resonance (CMR) and transthoracic echocardiography (TTE), and proceed to ICA and revascularization if possible. Blinded core laboratory interpretation will be performed for ICA, CCTA, PET/CT, CMR, and TTE. All participants will be followed up for 12 months. The primary endpoint is the improvement to the decrease of myocardial viability from baseline assessed with the use of PET/CT after 12-month follow-up.

**Conclusions:**

All of the patients are appropriately consented before enrolling in this study, which has been approved by the Ethics Committee. Results of SOS-COMEDY will be helpful to develop a strategy for single CTO patients.

## 1 Introduction

A chronic total occlusion (CTO) is defined as a complete coronary arterial obstruction with Thrombolysis In Myocardial Infarction (TIMI) flow grade of 0 for longer than 3 months.^[1]^ CTOs have been identified in up to 20% of all patients referred for diagnostic angiography.^[2]^ However, the CTO procedural success rate is 60% to 70%, much lower compared with non-CTO percutaneous coronary intervention (PCI) success rate of 98%.^[2,3]^ In the past, patients with a CTO were often managed conservatively or surgically rather than with PCI.^[4]^ The barriers that only 8% to 15% of patients with CTO face during PCI-based revascularization are numerous^[5–7]^: lower expectation of procedural success; higher perceived risk of complications; increased technical complexity; motivation; and training or catheter lab resources.^[8]^ Due to improvements in technique and observational evidence that successful CTO intervention is associated with symptomatic relief of angina, lower rates of subsequent myocardial infarction (MI) and coronary artery bypass graft (CABG), and survival improvement,^[9–16]^ there is growing interest in percutaneous treatment of CTO. Not only European but also American guidelines have assigned a class IIa (level of evidence B) recommendation for CTO PCI. However, there is currently no consensus on which strategy is much better. As a result, we divided patients into different groups using decision aid (DA) instead of randomization to protect the interests of patients as well as to increase compliance.

It is well known that the amount of viable myocardium is the best indicator of long-term cardiac event-free survival after cardiac intervention.^[17]^ Most CTO registries do not report preprocedural ischemic burden or myocardial viability. As such, the benefits of successful CTO PCI may be underestimated by the inclusion of patients with no viability or little ischemia that could not be expected to benefit from a successful procedure.

There are several diagnostic approaches used in current clinical practice for assessment of myocardial viability. Analysis of wall thickness or myocardial contraction, and evaluation of cardiac perfusion or metabolism can be assessed using the following modalities: transthoracic echocardiography (TTE), cardiac molecular imaging techniques [positron emission tomography (PET), single photon emission computed tomography (SPECT)], cardiac magnetic resonance (CMR), or coronary computed tomography angiography (CCTA). The combined devices multislice computed tomography scanners with PET (PET/CT) are now widely used in clinical practice. This combination allows for wider morphologic assessments: coronary calcium scoring and noninvasive coronary angiography may be added to myocardial perfusion/metabolic imaging if necessary.

In practice, it is difficult to accurately assess the impact of PCI and/or optimal medical therapy (OMT) alone on clinical outcomes due to inclusion of patients with heterogeneous coronary artery disease such as multivessel disease. Thus, patients with CTO PCI of a single coronary artery are an optimal study population to compare the relatively absolute impacts of PCI and OMT on clinical outcomes. Therefore, the aim of this study was to compare the improvement in the decrease of myocardial viability by PCI with that by OMT alone in patients with CTO of a single coronary artery in the drug-eluting stent (DES) era.

## 2 Methods

### 2.1 Design

To evaluate the change of myocardial viability in single CTO patients with successful PCI or with only OMT, we propose to conduct a prospective, nonrandomized multicenter trial using with a DA. The Ethics Committee in The First Affiliated Hospital of Dalian Medical University approved all study procedures. Patients and interventional cardiologists for this trial are to be recruited at 4 hospitals. All cardiologists have more than 10 years of PCI experience and perform CTO cases at least 50 cases each year.

### 2.2 Participants

**Figure 1:**
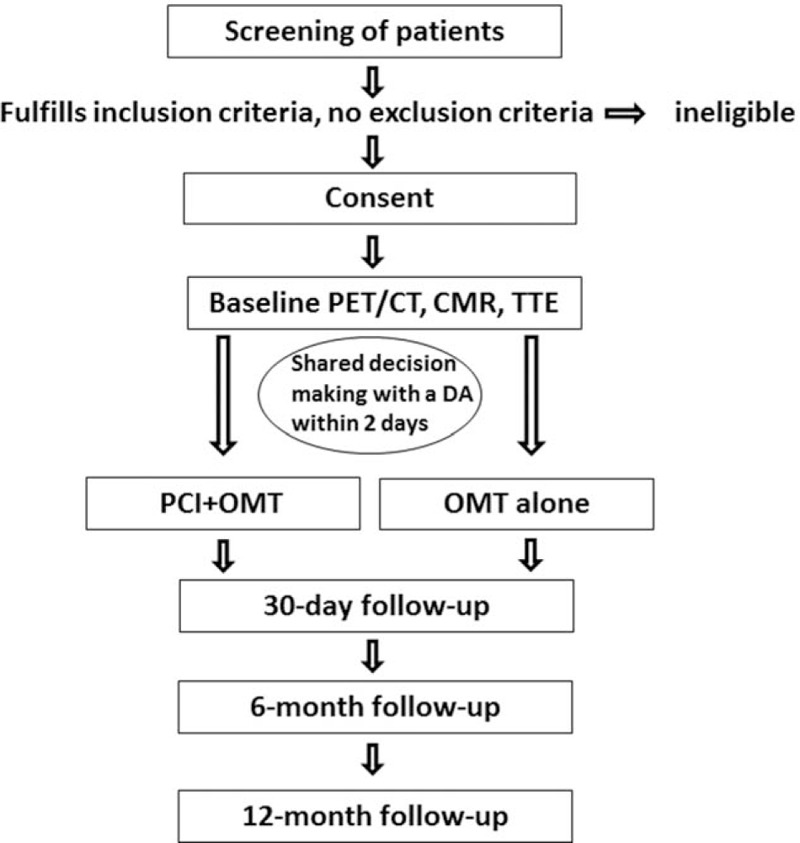
Study flow diagram outlining the study design.

Patients who satisfy all the inclusion criteria will be enrolled. Patients who meet any of the exclusion criteria will not be enrolled. The patients will give written informed consent to undergo PET/CT, CMR and TTE at baseline and at the 12-month follow-up, and participants will subsequently precede to invasive coronary angiography (ICA) and revascularization within 2 days of noninvasive tests (Fig. 1). Blinded core laboratory interpretation will be performed for ICA, CCTA, PET/CT, CMR, and TTE.

### 2.3 Inclusion criteria

Eligible patients include age 18 to 80, with a history of stable or unstable angina pectoris based on standard definitions for angina pectoris used in previous trials, whose left ventricular ejection fraction (LVEF) > 35% on TTE measurement, who is found single lesion occluding the coronary artery, who is with or without stenosis of other coronary arteries (≤ 50% diameter stenosis) detected by previous ICA or CCTA, who have a follow-up appointment with their clinician, and who are available for a clinic follow-up 12 months after enrollment.

#### 2.3.1 Exclusion criteria

The trial will exclude patients who have an acute Q-wave MI within the last 3 months, who have undergone revascularization in the nonculprit artery during the latest month, who are unsuitable for PCI, who are unable to tolerate dual antiplatelet treatment (DAPT), who have severe abnormal hematopoietic system (such as platelet count of < 100×10^9^/L or > 700×10^9^/L and white blood cell count of < 3×10^9^/L), who have active bleeding or bleeding tendency, who have severe coexisting conditions (such as severe renal insufficiency (GFR < 60 mL/min × 1.73 m^2^), severe hepatic dysfunction [elevated alanine transaminase (glutamic-pyruvic transaminase) or aspartate aminotransferase (glutamic-oxal acetic transaminase) level by more than 3-fold of the normal limitation], acute or chronic heart failure (NYHA III-IV), acute infectious diseases, immune disorders, malignancy), who have asthma, who have life expectancy < 12 months, who are pregnant or are planning pregnancy, who have drug allergies or contraindications to aspirin, clopidogrel, ticagrelor, statins, radiocontrast, anticoagulants, or stents, who are participating or planning to participate in another clinical trial during the same period, who have major learning barriers such as visual or hearing impairment or dementia that would compromise their ability to give written informed consent or use the decision aid.

## 3 Interventions

This study will be conducted in accordance with the Declaration of Helsinki and with the approval of the ethical committees of the 4 participating institutions. Recruitment commenced on October 20, 2015, and as of November 2017, 240 patients have been enrolled. The trial is registered under www.clinicaltrials.gov: NCT02767401.

## 4 Study definitions

A CTO was defined as a complete occlusion with TIMI flow grade 0 antegrade through the affected segment of >3 months’ duration in the opinion of the operator based on clinical features, angiographic features, and/or previous imaging. Procedural success was defined as < 50% residual stenosis with TIMI flow grade 3 antegrade. Failure was defined as failure to cross the occlusion or reduce obstruction to less than 50% in the target CTO.

### 4.1 Decision aid (DA)

**Figure 2:**
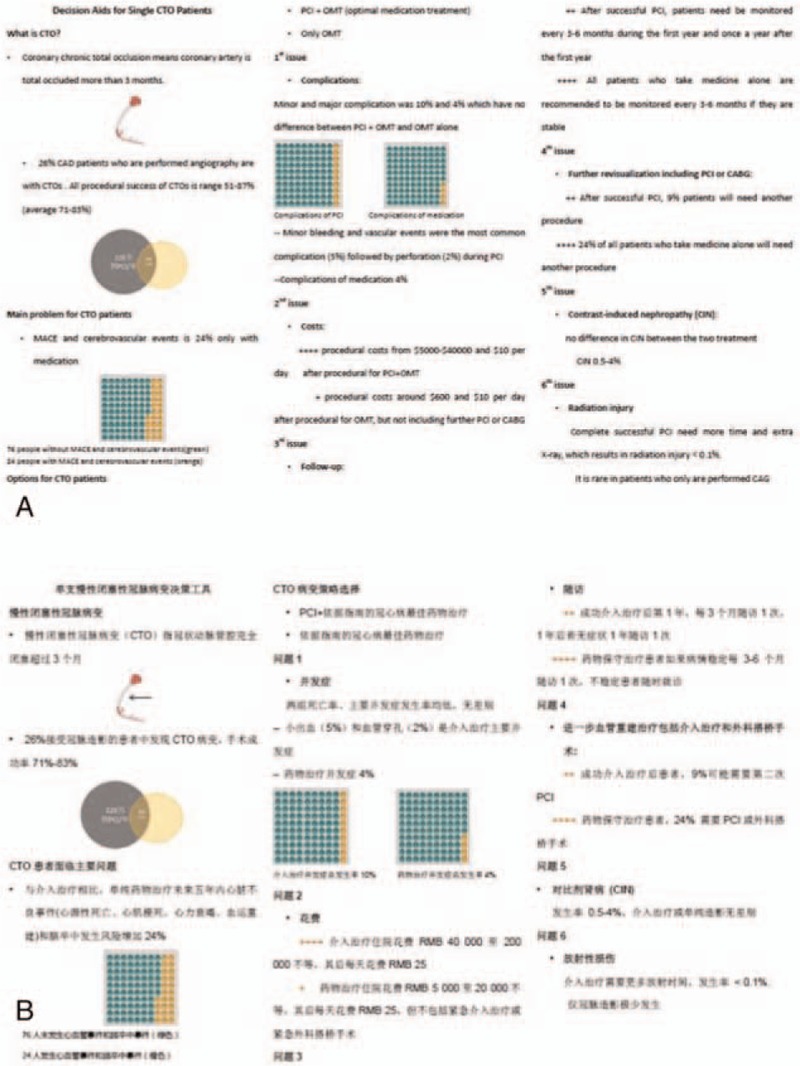
The pilot study of decision aid (DA) of SOS-comedy for patients with CTO lesions both in English (A) and Chinese (B) version. CTO = chronic total occlusion, SOS = success of opening single.

As shown in Fig. 2, in the pilot trial of DA, the major benefits and risks for patients using both strategies are listed. Only options with the most concerns will be presented in the final version of the questionnaire, including outcomes, complications, costs, follow-up requirement, further revisualization, contrast-induced nephropathy, and radiation injury. With the DA, the clinicians introduce all major benefits and risks if CTO patients choose PCI or medication using the words such as “during the next 5 years, among 100 patients like you, 5 patients have a risk of myocardial infarction…”.

### 4.2 Treatment details

At baseline, 3 months, 6 months, and 12 months recommendations for therapy are made to the physicians in line with guideline.^[18]^ The goal of antihypertensive therapy is to achieve a blood pressure of less than 140/80 mm Hg. The aim of antilipid therapy is to achieve levels of low-density lipoprotein (LDL) < 1.8 mmol/L. In the first instance, statin therapy will be initiated and then increased with the addition of a second agent if necessary. For patients with a BMI of > 25 kg/m^2^, the physicians are asked to refer the patient for dietary advice. Similarly, smokers are referred to the smoking cessation clinic. In the case of diabetics with raised blood sugar, the physicians are asked to measure HbA1c and to ensure that the patients’ subsequent therapy is tailored to achieve an HbA1c of less than 6.5 mg/dL. OMT included antiplatelet medication, β-blockers, rennin angiotensin system blockers, nitrates, calcium channel blockers, and aggressive lipid lowering therapy. The decision to pursue invasive treatment as well as site of access, type of stents, use of intravascular ultrasound, and use of glycoprotein IIb/IIIa receptor inhibitors was left to the discretion of the physician. Prior to PCI, all patients were pretreated with loading doses of aspirin (300 mg) and clopidogrel (300–600 mg) unless they were previously medicated with these antiplatelet agents. PCI was performed using contemporary techniques such as bilateral injections, specialized stiff hydrophilic wire with a tapered tip, and microcatheters in a retrograde approach, when available. All interventions and procedural anticoagulations were performed in accordance with current standard guidelines.^[19]^

### 4.3 Myocardial F-18 FDG PET/CT

Myocardial F-18 FDG PET/CT was performed within 2 days of each other. After an overnight fasting for at least 12 hours, an oral glucose of 10 to 50 g was given to the patients according to their serum glucose level. For diabetics, acipimox was administrated (500 mg; oral dose) before glucose loading. Insulin was intravenously administrated if the blood glucose level >9 mmol/L at 45 minutes after oral glucose administration with close monitoring of blood glucose. When the blood glucose level was appropriate, F-18 FDG (3 MBq/kg) was administered intravenously. Images were acquired 1 to 2 hours after tracer injection using a Biograph mCT 128 PET/CT scanner (Siemens Medical Solutions, Knoxville, TN) equipped with high-performance LSO PET crystals and a 128-slice CT. After a scout CT acquisition (120 kV, 10 mA) used for proper patients positioning, a CT transmission scanning (140 kV, 35 mA) was performed for attenuation correction and anatomical localization. Images were reconstructed using TrueX+TOFUltralHD reconstruction (21 subsets, 4 iterations). The Cedars software (Cedars-Sinai Medical Center, Los Angeles, CA) for phase analysis was applied on F-18 FDG PET/CT scans.

Nongated SPECT images were used for myocardial perfusion assessment by Quantitative Perfusion SPECT. Perfusion defect extent was expressed as a percentage of the whole myocardium area. Scar burden and myocardial viability were assessed by 2 experienced nuclear physicians. Segments with tracer uptake < 50% were considered transmural infarcted myocardium. F-18 FDG PET images were read in combination with rest SPECT images for myocardial viability assessment. The patterns of perfusion-metabolism mismatch and normal perfusion irrespective of F-18 FDG uptake signified viable myocardium whereas perfusion-metabolism match signified myocardial scar or nonviable tissue. The extent of viable myocardium and scar was expressed as percent of the LV area (%).

### 4.4 CMR scanning

CMR imaging was performed within 2 days of each other. CMR imaging will be performed on 3.0 T whole-body scanner (MAGNETOM Verio, A Tim System; Siemens Healthcare, Erlangen, Germany). A 32-element matrix coil was activated for data collection. All images will be acquired using phased array surface coils during mild expiration and electrocardiographic triggering. Cine images will be acquired in the 4-, 2-chamber and contiguous short axis views using a steady state-free precession (SSFP) sequence. A bolus of 0.05 mmol/kg of gadolinium injected at 5 mL/s through a dedicated right antecubital vein. Ten minutes after the first gadolinium injection; perfusion imaging was repeated. A 2-dimensional phase-sensitive inversion recovery breath-hold sequence was used for LGE imaging. This acquisition was performed for at least 8 minutes (but no longer than 30 minutes) after the last administration of gadolinium.

## 5 Endpoints

### 5.1 Primary endpoint

The primary study endpoint of the SOS-comedy trial is changed to myocardial viability from baseline assessed with the use of PET/CT system during 12-month follow-up.

### 5.2 Secondary endpoints

- Major adverse cardiac events (MACEs) including all-cause mortality, cardiac mortality, first or recurrent acute MI, recurrent angina, target lesion revascularization (TLR), heart failure, and rehospitalization at 30-day, 6-month, and 12-month follow-up;
- The rates of target vessel revascularization, TLR, and stent thrombosis at 30-day, 6-month, and 12-month follow-up;
- Stroke incidence;
- Changes to LVEF assessed with the use of CMR and TTE;
- Changes to myocardial infarct size, left ventricular mass (LVM), cardiac output (CO), stroke volume (SV), maximum left ventricular ejection rate, maximum left ventricular filling rate, and maximum slope assessed with the use of CMR;
- Changes to left ventricular end-diastolic diameter (LVEDd), left ventricular end-systolic diameter (LVESd), and cardiac systolic function (E/A, E’/A’, Ea/Aa, EDT) assessed with the use of TTE;
- The number of compliant patients which defined as those who take predefined percentage (100%) of the treatment;
- The total cost of medical care;
- Number of guidewires, balloons, and stents used in the procedure;
- The volume of contrast used during the procedure;
- Type of the first guidewire and the final guidewire to cross the proximal lesion and the new devices used in the procedures;
- The special techniques used in the procedures.

## 6 Safety monitoring

For the safety evaluation, the numbers and prevalence of adverse events including MACEs and stroke will be evaluated at each study visit. Stroke is defined as any new neurologic symptoms in association with signs of ischemia or hemorrhage in cranial computed tomography or magnetic resonance imaging.

## 7 Sample size calculation

The main goal of this study is to estimate the improvement of myocardial viability associated with different treatment strategies evaluated by PET/CT. Thank you for your kind comments. We set the sample size by 2 means. First, at the time of study design and commencement, no studies examined the effect of PCI and/or OMT on myocardial viability in single-vessel CTO. Therefore, it is difficult to calculate a target number of patients that should achieve the primary endpoint. For this reason, we consulted with statisticians and set sample size to 200 patients according to previous studies on CTO, which is more than previous studies and feasible to realize. Second, retrospective data indicates that about 80% patients could reach the improvement rate of 5% by PCI, which indicates significant. Approximately 20% patients treated with OMT only can be improved more than 0.5%. We hypothesize that 40% patients’ myocardial viability of both groups could be improved significantly. A total of 131 patients is needed to reject the null hypothesis of no difference between groups (2-sided test *χ*^2^ test, power 90%, α=0.05). Allowing for a 15% drop-out rate, 151 patients will be recruited. Since this is a multicenter study, the sample size was enlarged to 200 patients.

## 8 Statistical analysis

Normally distributed continuous variables were presented as the mean ± SD, variables with a skewed distribution as the median together with interquartile range (IQR). Categorical variables are expressed as percentages. Changes in data measured by PET/CT, CMR, and TTE will be summarized by mean, standard deviation, minimum, median, and maximum. Differences between groups, including baseline characteristics, will be compared using a *t* test, Wilcoxon rank-sum test, or *χ*^2^ test. The correlation between treatment strategies and viable myocardium will be analyzed by liner regression analysis.

For the safety evaluation, the number and prevalence of MACEs will be calculated. The Kaplan–Meier method was used to illustrate the timing of events during follow-up in relation to treatment strategies and statistical assessment was performed with the log-rank test. The prognostic value of treatment strategies was assessed by investigating its relation with the composite outcome in Cox proportional-hazards analyses.

## 9 Discussion and limitations

The last 5 years have been a significant increase in the number of publications focusing on coronary CTOs. The great majority of studies have compared successful with unsuccessful CTO PCI and only in recent years that the first trials specifically comparing CTO PCI with OMT have begun. The optimal treatment strategy for CTO patients is undetermined.

Several studies have shown that CTO-PCI is associated with clinical improvement.^[20]^ Cardona et al^[21]^ studied 29 patients with heart failure with reduced ejection fraction (HFrEF) and evidence of viability and/or ischemia within the territory supplied by the CTO, and improvement in ejection fraction, left ventricular end-systolic volume and ischemia burden was observed after CTO-PCI. Hwang et al^[22]^ compared clinical outcomes of PCI with those of OMT alone in patients with CTO of a single coronary artery. Four hundred thirty-five patients with CTO of a single coronary artery were analyzed. After a median follow-up duration of 47.6 months, MACEs were noted for 16 patients in the OMT group compared with 41 patients in the PCI group. After propensity-score matching, there were no significant differences between the OMT group and PCI group with respect to MACE frequency or cardiac death. However, the study did not routinely evaluate the amount of viable myocardium or ischemia of study patients using functional ischemia testing. One of the most critical steps in the management of the patients with obstructive coronary heart disease is to determine ischemic burden whether or not the patient is symptomatic. It is well known that stress radionuclide myocardial perfusion imaging (rMPI) may distinguish patients at high risk (more than 5% annual mortality rate) from those at intermediate risk (1% to 5% annual mortality rate) or low risk (<1% annual mortality rate).^[22]^ It was reported that the presence of extensive ischemia (extent of ischemia >10%), ischemia in multiple segments, ischemia in more than 1 coronary artery territory, LVEF < 45%, large fixed defects, transient or persistent left ventricular cavity dilatation and increased lung uptake of thallium on rMPI denotes high risk for increased risk of cardiac events.^[23–25]^ Safley et al^[26]^ showed that PCI in patients with CTOs significantly decreased the ischemic burden from 13.1% to 6.9%. In that clinical study most of the patients had moderate to severe ischemic burden before treatment. On the other hand, it was shown that there was improved survival following revascularization (PCI or CABG) only in the presence of moderate to severe baseline ischemic burden used by rMPI in coronary artery patients. The appropriate use criteria heavily emphasize ischemic burden and suggest that revascularization is appropriate in cases with large territories of ischemia, even without symptoms.^[27,28]^ Stuijfzand et al^[29]^ demonstrated that CTO patients with documented ischemia and viability showed significant improvement in stress MBF and a reduction of ischemic burden after successful percutaneous revascularization. However, the duration from PCI to follow-up imaging was 99 days, which may underestimate the effect of procedures. Second, the patients’ symptoms or quality of life before and after treatment were not sufficiently documented. Last, although the anatomical SYNTAX score is an important tool to establish the optimum revascularization approach in patients with complex coronary heart diseases including CTO, SYNTAX score II can better score than anatomical SYNTAX to reach the best treatment alternative. A combination of anatomical SYNTAX score with 3 simple clinical variables (age, creatinine, or creatinine clearance, LVEF) has been shown to contain most of the prognostic information in predicting mortality after PCI.^[30,31]^ From that point of view it should be better to use SYNTAX score II.

## 10 Conclusions

This study will be the first multicenter study to evaluate the effect of PCI and/or OMT on myocardial viability in patients with single-vessel CTO using combined PET/CT during long-term follow-up. The patients were divided after shared decision making process, which can protect the interests of patients as well as increase compliance. Furthermore, the study will show the effects of PCI and/or OMT strategies on the clinical outcomes in patients with CTO of a single coronary artery. Thus, SOS comedy will indicate the optimal treatment modality for single lesions resulting in CTO.

## Acknowledgments

The Study Design: Rongchong Huang, Xuchen Zhou, Xiantao Song, Shuzheng LYU, Victor M. Montori. The DA Design: Hargrave Ian, Rongchong Huang, Kasey R. Boehmer. The Performance of Percutaneous Coronary Intervention: Xiantao Song, Fei Yuan, Changjiang Ge, Rongchong Huang, Junjie Wang, Zhenguo Zheng, Wen Tian, Haishan Zhang, Zheng Huang, JianchengXiu, Xingwei Zhang. The Performance of TTE: Ya Yang, Yueli Wang, ShuangMeng, Hui Wang. The Performance of CMR: Yi He,Rui Wang, Zhiyong Li, Dianxiu Ning. The Performance of PET/CT: Xuemei Du, Xin Zhang, Hongzhi Mi. The Data Collection and Follow-up: Dongfeng Zhang, Kun Chen, Minjie Li, Wen Tian, Haishan Zhang, Zheng Huang, JianchengXiu, Dong Yang, Wenmin Liu. The Data Input: Dalian Ant Mobile Intelligent Health Management and Consulting Co., Ltd. The Data Analysis: Aaron L. Leppin, Hua Zhong, Na Xu, HuijuanZuo. The Data Monitoring Committee: Lei Zhong, Baolin Wu, Qi Li, Yalei Chen, Jianan Li. Manuscript Writing and Revision: Rongchong Huang, Dongfeng Zhang, Xiantao Song, Victor M. Montori, Aaron L. Leppin, Kasey R. Boehmer. The authors gratefully acknowledge the patients who participated in this study and the staff in the 4 centers who supported it.

## Author contributions

Rongchong Huang, Xiantao Song, Victor M. Montori, and Henry H. Ting designed the manuscript. Haishan Zhang, Wen Tian, Zheng Huang, and Xingwei Zhang participated in the intervention implementation. Dongfeng Zhang and Jian Wu collaborated in the design of statistical analyses. All the authors have revised and approved the final version.

**Conceptualization:** Rongchong Huang.

**Data curation:** Dongfeng Zhang, Jian Wu.

**Formal analysis:** Henry H. Ting.

**Investigation:** Jian Wu.

**Project administration:** Rongchong Huang, Haishan Zhang, Wan Tian, Zheng Huang, Xingwei Zhang, Junqing Yang.

**Supervision:** Lei Zhong.

**Writing – original draft:** Rongchong Huang, Xiantao Song.
